# Supplementary material for: OsGPAT3 Plays a Critical Role in Anther Wall Programmed Cell Death and Pollen Development in Rice
Source: Int J Mol Sci. 2018 Dec 12;19(12):4017. doi: 10.3390/ijms19124017 (PMC6321289; doi:10.3390/ijms19124017)
Supplement: Supplementary file 1 [file ijms-19-04017-s001.pdf]

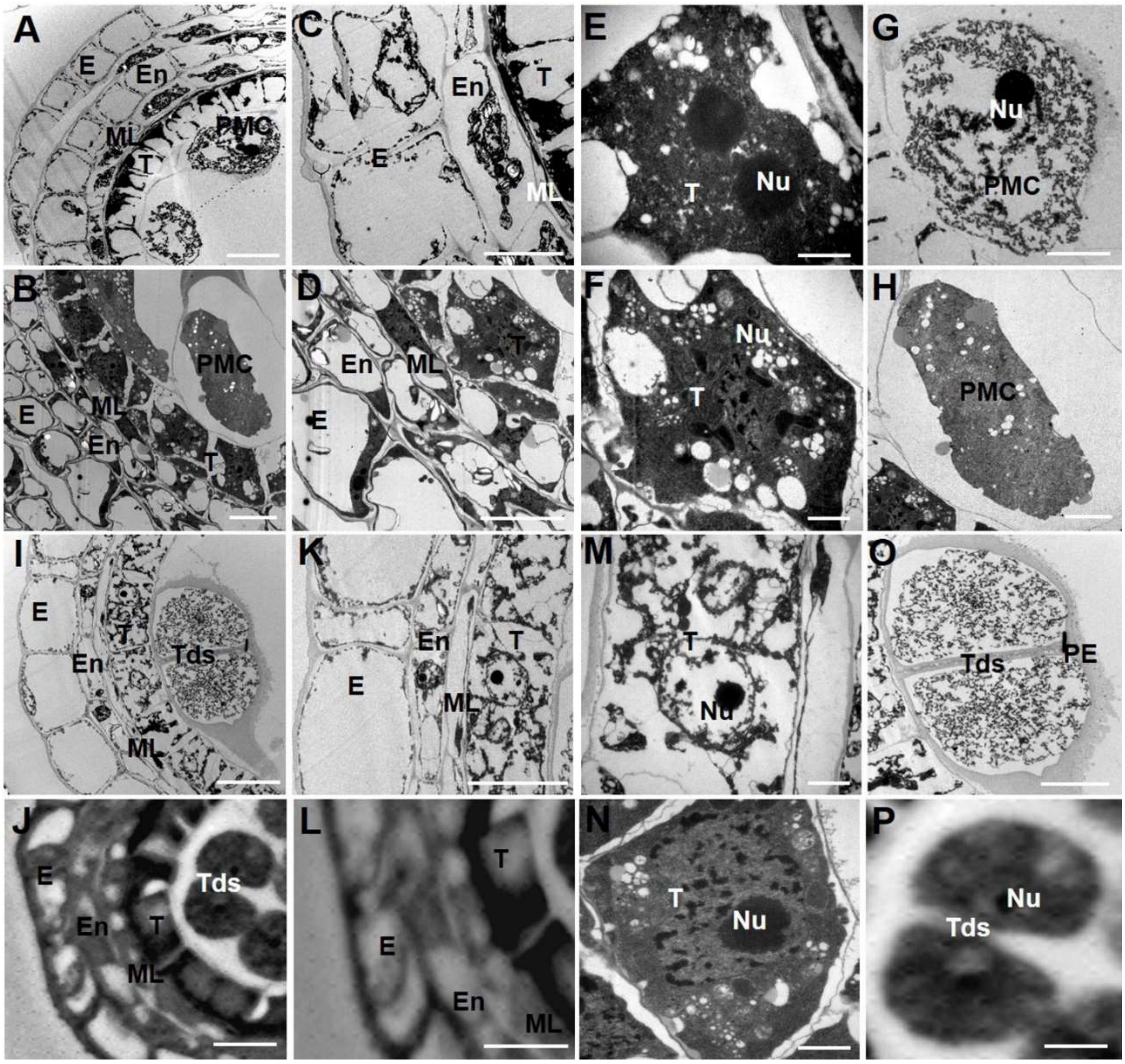

Figure S1. Transmission electron microscopy analysis of anthers in wild type and *osgpat3-2-2* mutant at stage 8. The transverse sections of the wild-type (**A**,**C**,**E**,**G**,**I**,**K**,**M**,**O**) and *gpat3-2* (**B**,**D**,**F**,**H**,**J**,**L**,**N**,**P**) anthers at stage 8a (**A**–**H**) and stage 8b (**I**–**P**) are compared. (**A**,**B**,**I**,**J**) Anthers of the wild type (**A**,**I**) and *osgpat3-2* (**B**,**J**), showing the four layers of the anther wall with pollen mother cell at stage 8a and tetrads at stage 8b. (**C**,**D**,**K**,**L**) The four layers of the anther wall of the wild type (**C**,**K**) and *osgpat3-2* (**D**,**L**). (**E**,**F**,**M**,**N**) Higher magnification of the tapetum cells showing Ubisch body of the wild type (**E**,**M**) and *osgpat3-2* (**F**,**N**). (**G**,**H**) Pollen mother cell of the wild type (**G**) and *osgpat3-2* (**H**) at stage 8a. (**O**,**P**) Tetrads of the wild type (**O**) and *osgpat3-2* (**P**) at stage 8b. The arrows in (**E**,**F**,**M**,**N**) indicate an Ubisch body (Ub). E, Epidermis; En, Endothecium; ML, Middle Layer; Nu, Nucleus; PE, Priexine; PMC, Pollen Mother Cell; T, Tapetum; Tds, Tetrads. Bars = 10 μm in (**A**,**B**,**I**,**J**); 5 μm in (**C**,**D**,**J**,**K**); 0.5 μm in (**E**,**F**,**M**,**N**), and 2 μm in (**G**,**H**,**O**,**P**).

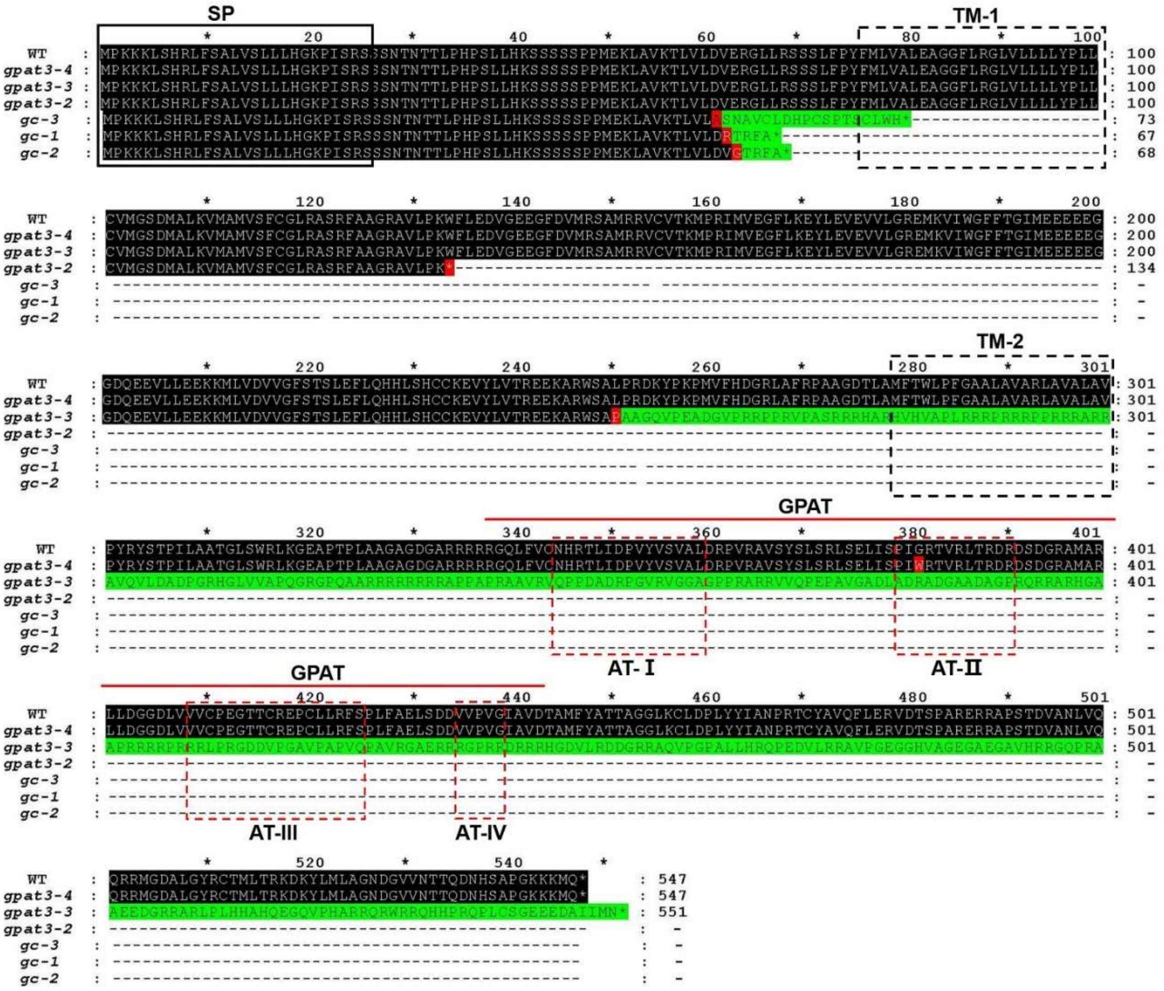

Figure S2. Alignments of OsGPAT3 sequence between the wild type (Zh8015, WT), three CRISPR/Cas9-induced mutants, and two allelic mutants. Analysis performed with DNASTAR-Lasergene v6/Megalign, and GENEDOC software. The signal peptide (black box), the conserved phospholipid/glycerol acyltransferase domain (GPAT, red line), transmembrane region (black dotted box), and four conserved acyltransferase motifs are indicated above the alignment (AT-I, AT-II, AT-III, and AT-IV, marked in red dotted box) were indicated by the BLAST search program (http://www.ncbi.nlm.nih.gov/blast/). Identical sequences, mutation sites, and different sequences after mutation of amino acid residues are shaded black, red, and green, respectively.

Table S1. Segregation analysis of the *gpat3-2* allele.

Combination	Seed-Setting Rate of F_1	F_2		χ^2(3:1)	$\chi^2_{0.05}$
		No. of Wild Type Plants	No. of Mutant Plants		
gpat3-2/Zh8015	76.75	289	91	0.17	3.84
gpat3-2/02428	82.31	472	138	1.71	

Table S2. Inferred function of candidate genes in the *gpat3-2* locus on rice Chr. 11.

Gene Number	Putative Function	Biological Process Involved	Specific Expression Tissue of Homologs in *Arabidopsis*.t
LOC_Os11g45390	von Willebrand Factor Type A Domain Containing Protein	Root Development	Root Meristem, Shoot Stem Cells, Root Quiescent Center
LOC_Os11g45400	Glycerol-3-Phosphate Acyltransferase	Anther Development and Pollen Formation	Seedlings, Leaves, Shoots, Panicles and Anthers, Siliques
LOC_Os11g45410	Tetratricopeptide Repeat (TPR)-like Superfamily Protein	---	Guard Cell, Shoot Apex

Table S3. Primer sequences used in this study.

Primer	Forward Primer (5′-3′)	Reverse Primer (5′-3′)	Purpose
RM27172	GAAAGAAGGGATGTCTTGCATGAGG	GAACATCCTAACCACGTCGGAAGC	Linkage analysis
RD1110	GTCAGAGGAGTCGAATACG	ACCAACCAAACACCTAAAA	
RM27273	ATGCGTTTGCCGTGAAGAAAGG	GTCTGGGCCCACATGTCAATAGC	
RM27326	ATCAACGAGTACGCAACAGTCC	TCCTGTCTCTTCACATCCTAATCG	
ZH-3	TGTCGAGGTTCTTGCAGACG	GACGTCGACCACTCCGAA	Fine mapping
ZH4	GTTCTCCGAGGGGGTTGTTC	ATACGCAAAAACTCCTGCCC	
ZH5	CTAAACTCGTCTATCACCATCGTTC	CCATCGTATATTACCCATGAACAT	
ZH-6	GCGGATTCGACCAACAGTA	ATCAAGTAAACATCTTAACACAACT	
ZH-7	GATCGGCCGAATTCCACAAT	ACACCAACCGGAAACTAAAGA	
ZH-8	GGCGACAATGTAACCGTCAG	ATCAATGATTGCACGATCAGC	Sequencing
ZH-9	ATTTTATTTCCCCAACCACTTTT	CAATGACTTAACATCAATTCTTAGCA	
CAPS-1	TTGAAGGTCATGGCAATGGT	CAAGCAACACCTCCTCTTGA	Enzyme Degistion
CAPS-2	TACCCATTGCTATGTGTCATGG	AAGAACCCCCAAATCACCTT	
GP-COM-*BamH* I	CGGTACCCGGGGATCCGGGACAAGACGGTCCCAATC	CGACTCTAGAGGATCCTAGTGGCATGTGGATTGCATTG	Complementation vector construction
GP-Cas-*Aar* I	AGATGATCCGTGGCACTAGTACTCGACGTCGAAGGCGGGTTTTAGAGCTATGC	GCATAGCTCTAAAACCCGCCTTCGACGTCGAGTACTAGTGCCACGGATCATCT	CRISPR/Cas9 vector construction
Cas-Seq	GGCTGTTCTCTGCCTTGGTAT	TCTTTGTCACACACACCCTCC	Sequencing
OsGPAT3	GCAGAGGAGGATGGGCG	TGGTTGTCTTGGGTGGTGTT	qPCR
LOC_Os05g38350	GCGATCCTTCTTGCCCTTC	GGAACGGAGGGCATTTGTG	
LOC_Os10g42720	GCCATAATGTTGTTCTGATGTC	CTTCAGACTACGAGTATTTGC	
